# Dimerization of Translationally Controlled Tumor Protein Is Essential For Its Cytokine-Like Activity

**DOI:** 10.1371/journal.pone.0006464

**Published:** 2009-07-31

**Authors:** Miyoung Kim, Hyun Jung Min, Hee Yeon Won, Heejin Park, Ji-Chul Lee, Heung-Woo Park, Junho Chung, Eun Sook Hwang, Kyunglim Lee

**Affiliations:** 1 College of Pharmacy, Center for Cell Signaling Research and Drug Discovery Research, Ewha Womans University, Seoul, Korea; 2 Abxign, Seoul, Korea; 3 Division of Allergy and Clinical Immunology, Seoul National University Hospital, Seoul, Korea; 4 College of Medicine and Cancer Research Institute, Seoul National University, Seoul, Korea; Centre National de la Recherche Scientifique, France

## Abstract

**Background:**

Translationally Controlled Tumor Protein (TCTP) found in nasal lavage fluids of allergic patients was named IgE-dependent histamine-releasing factor (HRF). Human recombinant HRF (HrHRF) has been recently reported to be much less effective than HRF produced from activated mononuclear cells (HRFmn).

**Methods and Findings:**

We found that only NH_2_-terminal truncated, but not C-terminal truncated, TCTP shows cytokine releasing activity compared to full-length TCTP. Interestingly, only NH_2_-terminal truncated TCTP, unlike full-length TCTP, forms dimers through intermolecular disulfide bonds. We tested the activity of dimerized full-length TCTP generated by fusing it to rabbit Fc region. The untruncated-full length protein (Fc-HrTCTP) was more active than HrTCTP in BEAS-2B cells, suggesting that dimerization of TCTP, rather than truncation, is essential for the activation of TCTP in allergic responses. We used confocal microscopy to evaluate the affinity of TCTPs to its putative receptor. We detected stronger fluorescence in the plasma membrane of BEAS-2B cells incubated with Del-N11TCTP than those incubated with rat recombinant TCTP (RrTCTP). Allergenic activity of Del-N11TCTP prompted us to see whether the NH_2_-terminal truncated TCTP can induce allergic airway inflammation in vivo. While RrTCTP had no influence on airway inflammation, Del-N11TCTP increased goblet cell hyperplasia in both lung and rhinal cavity. The dimerized protein was found in sera from allergic patients, and bronchoalveolar lavage fluids from airway inflamed mice.

**Conclusions:**

Dimerization of TCTP seems to be essential for its cytokine-like activity. Our study has potential to enhance the understanding of pathogenesis of allergic disease and provide a target for allergic drug development.

## Introduction

Translationally controlled tumor protein (TCTP), also variously known as IgE-dependent histamine-releasing factor (HRF) [1], p23/p21 [2], [3], and fortilin [4], is distributed in all normal cell types. It exhibits a high degree of homology among various species, suggesting that TCTP may play an essential role in cellular processes [5]. Many studies demonstrated that TCTP is a multifunctional protein [6]. TCTP was reported to be involved, extracellularly, in human allergic response as an HRF [1] and intracellularly, as a microtubule-stabilizing protein [7], as an antiapoptotic protein [4], and in protein synthesis as a guanine nucleotide dissociation inhibitor [8]. We reported recently that TCTP also acts as a suppressor of Na,K-ATPase [9].

Extracellularly TCTP appears outside of macrophages [10] and activated mononuclear cells [11], in bronchoalveolar lavage fluids (BALF) from patients with eosinophilic pneumonia and asthma [12], in nasal washings [13], and in skin blister fluids during the late allergic reaction [14]. Human recombinant HRF (HrHRF) directly stimulates the secretion of histamine, IL-4, and IL-13 from a subpopulation of basophils, and also enhances their secretion from all IgE-bearing basophils activated by anti-IgE Ab [15]. HrHRF stimulates the secretions of IL-8 and GM-CSF in primary cultures of human bronchial epithelial cells and human bronchial epithelial cell line, BEAS-2B [12].

HrHRF has been recently reported to be much less effective than HRF produced from activated mononuclear cells (HRFmn). Also, presence of HRFmn could not be demonstrated in serum when an HrHRF-specific ELISA assay was used [16]. These findings suggest that extracellular TCTP exhibiting cytokine-like activity *in vivo* might be a modified form of intracellular TCTP. We hypothesized that the observed differences in the activity of HRFmn and HrHRF might arise from modifications occurring in TCTP in the allergic environment. In this study, we tested this hypothesis by characterizing the active forms of TCTP and examining the conformational differences between the modified and the unmodified forms of TCTP. We also investigated whether the modified form of TCTP could induce allergic inflammation in vivo and whether it is present in sera from allergic patients and in bronchoalveolar lavage fluids from airway inflamed mice.

## Results

### NH_2_-terminal truncated RrTCTP increases IL-8 and GM-CSF secretion from BEAS-2B cells

**Figure 1:**
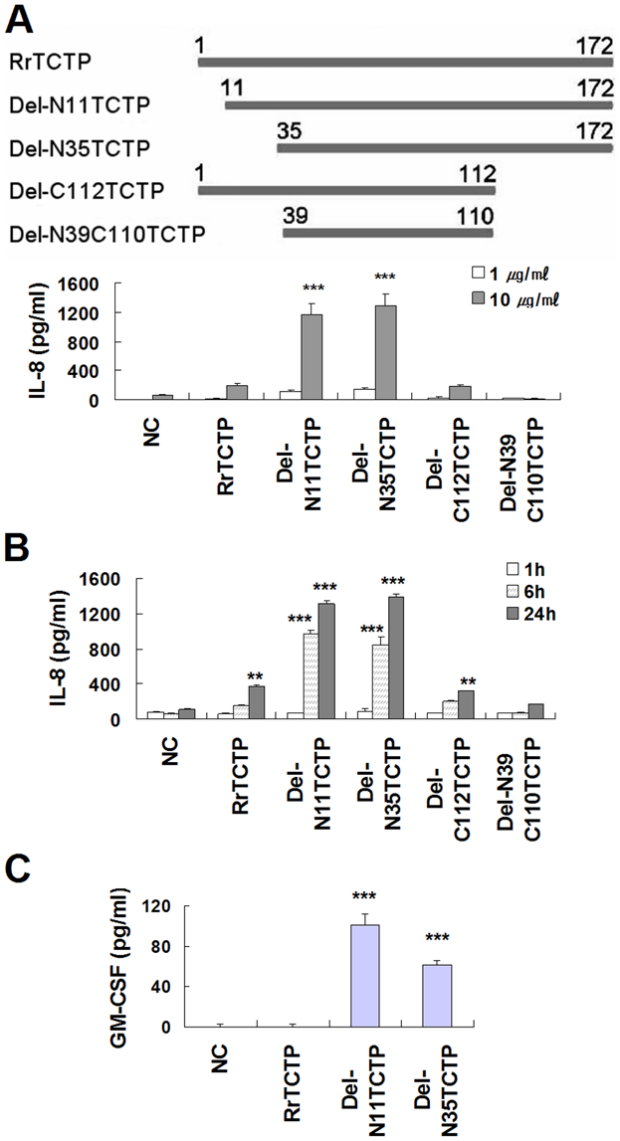
NH_2_-terminal truncated RrTCTPs show increased activity in BEAS-2B cells. (A) BEAS-2B cells were treated with 1 or 10 µm/ml of RrTCTP or various RrTCTP derivatives for 48 h. The resulting IL-8 in supernatant was analyzed by ELISA (*** *p*<0.001, significantly different from the NC value, n = 3). (B) 10 µm/ml of RrTCTP or RrTCTP derivatives were added to BEAS-2B cells and incubated for 1, 6, or 24 h (** *p*<0.01, *** *p*<0.001, significantly different from the NC value, n = 3). (C) 10 µm/ml of RrTCTP or RrTCTP derivatives were added to BEAS-2B cells and incubated for 48 h. The resulting GM-CSF in supernatant was analyzed by ELISA (*** *p*<0.001, significantly different from the NC value, n = 3).

Since endogenous proteases from neutrophils [17] and mast cells [18] are present in sites of inflammation, we wondered if TCTP acquires its cytokine-like activity following partial proteolysis by these proteases. To answer this question, we designed five rat TCTP constructs including a rat recombinant TCTP (RrTCTP) construct and four deletion constructs: Del-N11TCTP (residues 11–172), Del-N35TCTP (residues 35–172), Del-C112TCTP (residues 1–112), and Del-N39C110TCTP (residues 39–110) (Figure 1A). RrTCTP and the RrTCTP deletion derivatives were tested for their ability to stimulate the secretion of IL-8 [12] from BEAS-2B bronchial epithelial cells. When BEAS-2B cells were treated with RrTCTP, IL-8 release was stimulated in a dose- and time-dependent fashion (Figure 1, A and B). Under the same experimental condition, Del-N11TCTP and Del-N35TCTP stimulated IL-8 release about 4–9 fold better than RrTCTP; Del-C112TCTP stimulated IL-release at about the same level as RrTCTP; Del-N39C110TCTP did not show any stimulatory activity, and this may be due to an alteration in its tertiary structure by excessive deletion. The level of GM-CSF secretion was somewhat lower than that of IL-8 secretion in BEAS-2B cells. We could measure GM-CSF secretion by Del-N11TCTP and Del-N35TCTP derivatives, but not by RrTCTP (Figure 1C). These results suggest that NH_2_-terminal truncation increases the cytokine-like activity of RrTCTP.

### NH_2_-terminal truncated RrTCTP enhances allergic response in T cells

**Figure 2:**
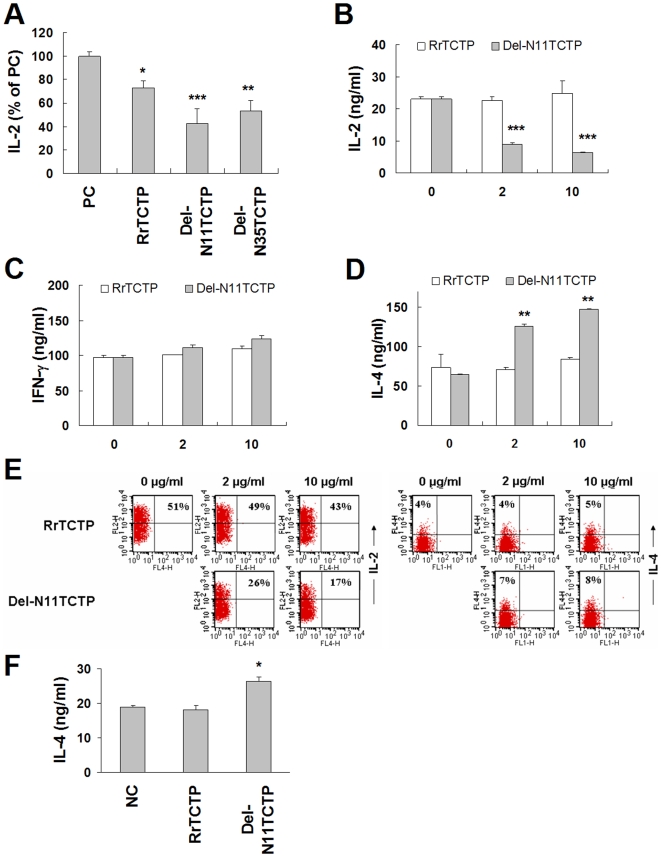
NH_2_-terminal truncated RrTCTPs augment inflammatory responses in T cells. (A) Jurkat cells were incubated with 10 µm/ml of RrTCTP or RrTCTP derivative for 4 h, and further stimulated with 10 µm/ml PMA and 125 µm/ml ionomycin for 20 h. IL-2 level in each supernatant was measured by ELISA. IL-2 release by 10 µm/ml PMA and 125 µm/ml ionomycin (positive control, PC) was expressed as 100 % (* *p*<0.05, ** *p*<0.01, *** *p*<0.001, significantly different from the PC value, n = 3). (B-D) Isolated CD4^+^ T_H_ cells were stimulated with anti-CD3 (1 µg/ml) and anti-CD28 (1 µg/ml) antibodies for 48 h, and 0 (without protein), 2 or 10 µm/mlof RrTCTP or Del-N11TCTP for 48 h. IL-2 or IFN-γ in supernatant were analyzed by ELISA (** *p*<0.01, *** *p*<0.001, significantly different from the ‘0 (without protein)’ value). (E) Purified CD4^+^ T_H_ cells were incubated with anti-CD3 and anti-CD28 antibodies and 2 or 10 µm/ml of RrTCTP or Del-N11TCTP for 72 h, and additionally stimulated with PMA and ionomycin for 4 h. Cells were treated with monensin for 2 h, and labeled with PE-IL-2 Ab or APC-IL-4 Ab. Cells were analyzed by FACS Calibur and CellQuest. (F) Isolated CD4^+^ T_H_ cells were differentiated into T_H_ 2 cells in the presence of IL-4 (10 µm/ml) and anti-IFN-γ (5 µg/ml) and re-stimulated. 10 µm/ml of RrTCTP or Del-N11TCTP were added the culture media every other day. The level of IL-4 was determined using ELISA (* *p*<0.05, significantly different from the NC value).

TCTP is known to inhibit the release of IL-2 in human peripheral blood T cells and Jurkat T cells [19]. Because Del-N11TCTP and Del-N35TCTP showed increased cytokine releasing activity in BEAS-2B cells, we further evaluated their activity in T cells. Jurkat T cells produce IL-2 when stimulated by PMA/calcium ionophore [20]. First, we examined the activation profile of our cells by PMA/ionomycin. IL-2 secretion was stimulated in a dose-dependent manner between 50–500 µm/ml of ionomycin in the presence of PMA (data not shown), and we used 125 µm/ml of ionomycin in additional experiments. Cells were preincubated in growth media with or without 10 µm/ml of RrTCTP, Del-N11TCTP, or Del-N35TCTP for 4 h, followed by 20 h incubation with PMA/ionomycin (Figure 2A). The inhibition pattern of IL-2 release correlated with activation pattern of IL-8 release: Del-N11TCTP was the most active. While RrTCTP suppressed 26.8±5.7% of IL-2 secretion, Del-N11TCTP and Del-N35TCTP suppressed 57.6±12.4% and 46.5±9.0% of IL-2 secretion in Jurkat T cells stimulated with PMA/ionomycin, respectively.

We also examined the effect of TCTP on cytokine production in murine CD4^+^ T_H_ cells upon TCR stimulation. In accordance with previous findings, Del-N11TCTP, but not RrTCTP, substantially suppressed IL-2 production in CD4^+^ T_H_ cells stimulated with anti-CD3 and anti-CD28 antibodies for 48 h (Figure 2B). Furthermore, the level of IL-4 increased by PMA and ionomycin was augmented in the presence of Del-N11TCTP, but not RrTCTP (Figure 2D), while IFN-γ was not changed by either RrTCTP or Del-N11TCTP (Figure 2C). Also, intracellular cytokine staining revealed decreased IL-2 and increased IL-4 secretion by Del-N11TCTP (Figure 2E). IFN-γ and IL-4 are the main cytokines in T_H_1 and T_H_2 cells, respectively [21]. Cross-inhibition of T_H_ cell differentiation by IFN-γ and IL-4 is well known [22]. Abnormal activation of T_H_2 cells causes allergic disorders [23]. When the cells differentiate into IL-4-producing T_H_2 cells, IL-4 production was significantly increased by Del-N11TCTP, but not by RrTCTP (Figure 2F). These results suggest that Del-N11TCTP, but not RrTCTP increases allergic cytokine IL-4 in CD4^+^ T_H_ cells, thereby promoting allergenic activity.

### NH_2_-terminal truncated RrTCTP forms intermolecular disulfide bridge

**Figure 3:**
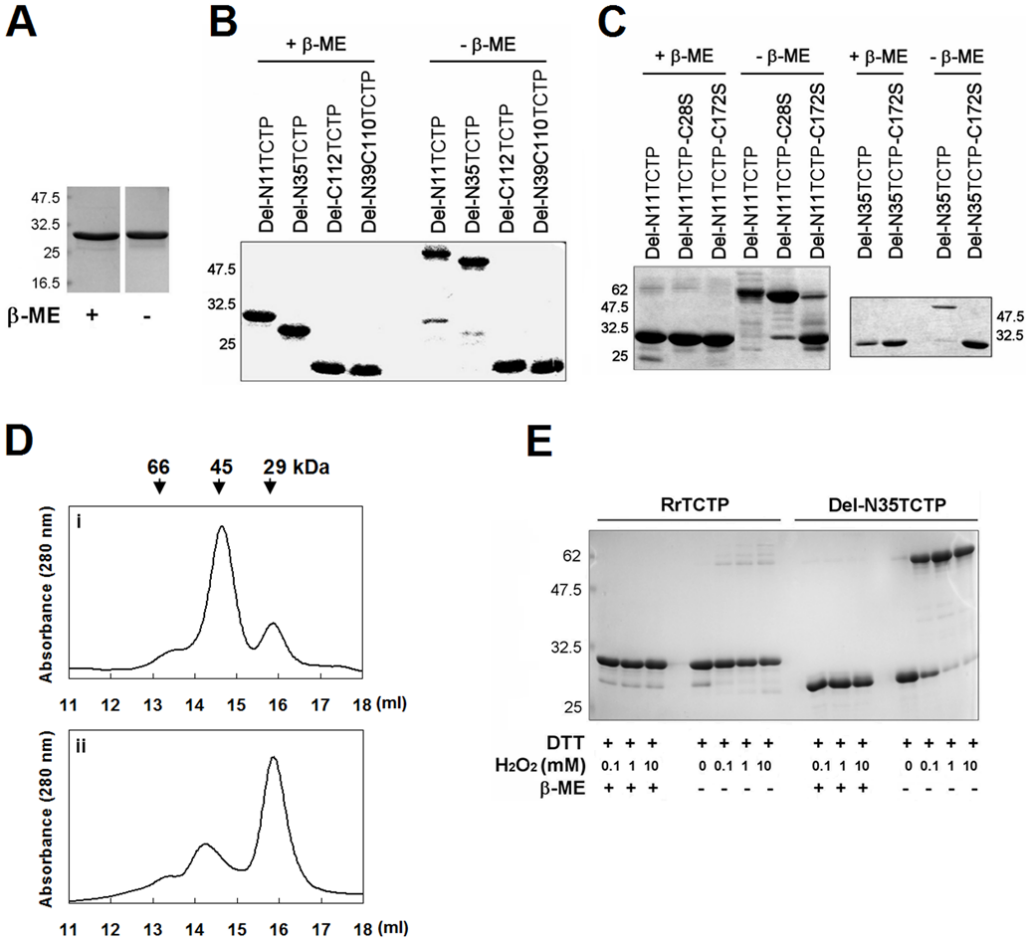
NH_2_-terminal truncated RrTCTP forms dimer by intermolecular disulfide bond. (A) RrTCTP was boiled with or without reducing agent and subjected to SDS-PAGE. (B) NH_2_-terminal or COOH-terminal truncated RrTCTPs were run in the presence or absence of β-ME on a 15% gel, and stained with coomassie brilliant blue. (C) Del-N11HRF, Del-N35HRF, and their mutants were analysed by reducing or non-reducing 15% SDS-PAGE. (D) Del-N35TCTP (i) and Del-N35TCTP-C172S (ii) were loaded onto Superdex 200 HR 10/30 gel filtration column (Amersham Pharmacia Biotech). The column was equilibrated and eluted with 50 mM sodium phosphate, pH 7.0 containing 150 mM NaCl. The eluting positions were calibrated with the molecular size marker (Sigma) and the peak positions of molecular mass standards are indicated by vertical arrow. (E) Completely reduced RrTCTP or Del-N35TCTP were transferred to 50 mM Tris, pH 7.5, 1 mM EDTA, and then oxidized with 0.1, 1, or 10 mM H_2_O_2_. Resulting products were analysed by reducing and non-reducing SDS-polyacrylamide gels.

To test whether the higher activity of the truncated protein is due to altered conformations not present in the full-length protein, we compared the mobility of these proteins in reducing and non-reducing SDS-polyacrylamide gels (Figure 3, A and B). We found that NH_2_-terminus containing proteins were all detected at molecular sizes corresponding to a monomer, regardless of the presence or absence of β-mercaptoethanol (β-ME), while the NH_2_-terminal truncated forms showed mobility shifts corresponding to a dimer under non-reducing conditions, which suggests that NH_2_-terminal truncated proteins form dimers probably through intermolecular disulfide bonds.

TCTP contains two cysteine residues, Cys^28^ and Cys^172^. To identify the residue forming disulfide bonds, we analyzed three mutants, Del-N11TCTP-C28S, Del-N11TCTP-C172S, and Del-N35TCTP-C172S by SDS-PAGE (Figure 3C). Under non-reducing conditions, Del-N11TCTP, Del-N35TCTP, and Del-N11TCTP-C28S electrophoresed at the apparent molecular weight corresponding to the dimer. Del-N11TCTP-C28S also showed an additional minor band corresponding to the monomer. Del-N11TCTP-C172S and Del-N35TCTP-C172S were detected at positions corresponding to the monomer, with Del-N11TCTP-C172S also showing an additional minor band corresponding to the dimer. These results suggest that although both Cys^28^ and Cys^172^ seem to be involved in the formation of intermolecular disulfide bond, Cys^172^ is the main site of dimerization.

There are several reports that TCTP homologues form dimeric structures [24]–[26]. To elucidate the oligomeric structure of TCTP, the apparent molecular weights of Del-N35TCTP and Del-N35TCTP-C172S were determined using size exclusion column chromatography (Figure 3D). Del-N35TCTP showed two peaks, a major peak of approximately 45 kDa corresponding to the dimeric form and a minor peak of about 29 kDa corresponding to the monomeric form (Figure 3D-i). Del-N35TCTP-C172S also showed two peaks, but the major peak corresponded to the monomeric form (Figure 3D-ii).

We also tested whether the NH_2_-terminal truncated RrTCTP can be dimerized by oxygen species such as H_2_O_2_. RrTCTP and Del-N35TCTP were completely reduced with 20 mM DTT, and then oxidized by increasing concentrations of H_2_O_2_ (Figure 3E). Whereas RrTCTP showed only minimal fraction of dimers, all the Del-N35TCTP migrated in the dimer position in non-reducing SDS-polyacrylamide gels in the presence of H_2_O_2_. This implies that only the NH_2_-terminal truncated TCTP can be modified by reactive oxygen species at sites of inflammation.

### Dimerization of RrTCTP by intermolecular disulfide bond is critical to its activity

**Figure 4:**
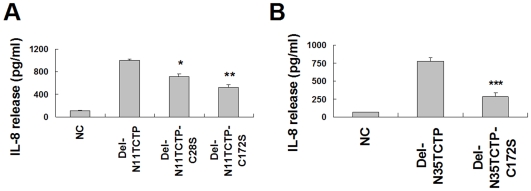
Dimerization by disulfide bond is essential for TCTP activity. (A, B) Secretion of IL-8 from BEAS-2B cells was measured after incubation with 5 µm/ml of Del-N11TCTP, Del-N35TCTP or their mutants for 24 h (* *p*<0.05, **p<0.01, ***p<0.001, significantly different from their wild-type value, n = 3).

In order to determine whether disulfide linkages of NH_2_-terminal truncated RrTCTP are critical for the cytokine-like activity, we compared IL-8 secretions stimulated by wild-type and mutant proteins. Both Del-N11TCTP-C172S and Del-N35TCTP-C172S exhibited reduced ability for IL-8 secretion (52% and 36% of wild-type, respectively) from BEAS-2B cells, indicating that disulfide bonds are important for the cytokine-like activity of RrTCTP (Figure 4, A and B). Del-N11TCTP-C172S caused more IL-8 release than Del-N35TCTP-C172S. This is probably due to the presence of a small amount of the dimer form of Del-N11TCTP-C172S, disulfide bonded through Cys^28^. Cys^28^ substitution slightly affected the secretion of IL-8 and the activity of Del-N35TCTP that does not contain Cys^28^ is slightly lower than that of Del-N11TCTP. Interestingly, Del-N35TCTP-C172S, which does not contain Cys^28^ and Cys^172^ is about two fold more active than RrTCTP (Figure 1B). This is probably due to the minor presence of dimer as seen in Figure 3D-ii. These results suggest that Cys^172^ may play a major role in the cross-linking of TCTP by disulfide bond and that the enhanced biological activity of the truncated RrTCTP may be due to the dimerization.

### Dimerization of TCTP, not truncation, is essential for the cytokine-like activity of TCTP

**Figure 5:**
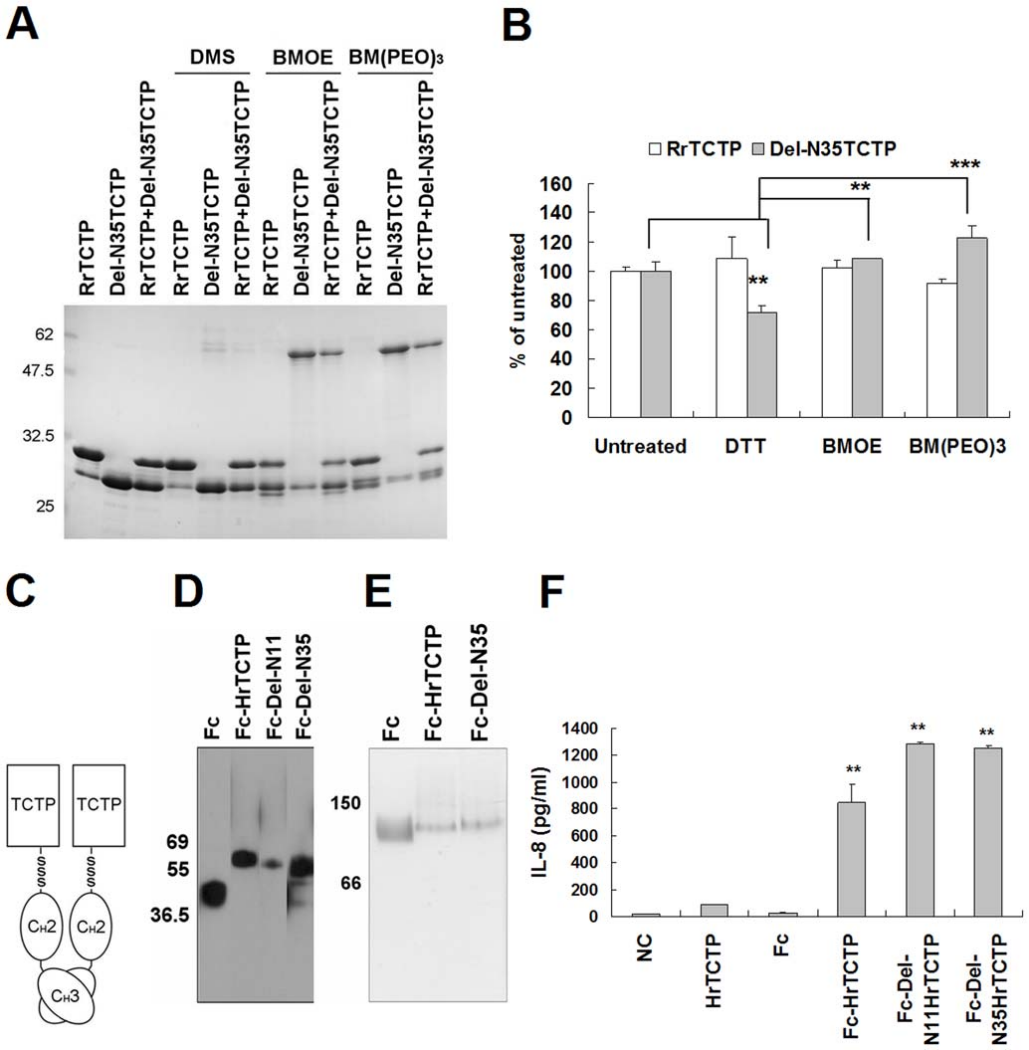
Dimerization of TCTP is necessary for the cytokine-like activity of TCTP. (A, B) RrTCTP and Del-N35TCTP were irreversibly cross-linked by DMS, BMOE, or BM(PEO)_3_. Proteins were completely reduced with 20 mM DTT before incubating with BMOE or BM(PEO)_3_. Crosslinker-treated RrTCTP, Del-N35TCTP, and their 1/2∶1/2 molar mixture were analysed by reducing SDS-PAGE. And, the secretion activities of IL-8 by DTT, BMOE, or BM(PEO)_3_-treated RrTCTP or Del-N35TCTP were compared using BEAS-2B cells (** *p*<0.01, ***p<0.001, significantly different from untreated Del-N35TCTP or DTT-treated Del-N35TCTP value, n = 3). (C) Schematic diagram of Fc-fused human recombinant TCTP (Fc-HrTCTP). (D) Fc fusion proteins were analyzed by Western blotting with HRP conjugated anti-rabbit IgG. (E) Fc fusion proteins were analyzed using native gel. The apparent molecular weights were confirmed by the molecular size marker (Sigma). (F) The release activities of IL-8 by indicated protein were compared in BEAS-2B cells (** *p*<0.01, significantly different from Fc value, n = 3).

To further characterize the active form of TCTP, we performed chemical crosslinking experiments using three irreversible crosslinkers, DMS (dimethyl suberimidate· 2HCl), BMOE (bis-maleimidoethane), and BM(PEO)_3_ (1, 11-bismaleimidotriethylene glycol). When BMOE or BM(PEO)_3_ was added, Del-N35TCTP formed dimer regardless of the length of spacer arm of crosslinkers in reducing SDS-PAGE (Figure 5A). Interestingly, a part of RrTCTP formed an intramolecular link by these reagents. Since only RrTCTP monomers and Del-N35TCTP dimers, but not intermediate products, were detected in the mixture of RrTCTP and Del-N35TCTP, it appears that Del-N35TCTP and RrTCTP can not be cross-linked each other. To test if these proteins could form dimers through interactions other than disulfide bond formation, we used DMS, an irreversible crosslinker for primary amines. RrTCTP and Del-N35TCTP did not form oligomers, implying that Del-N35TCTP may form dimers only through disulfide bonds. We measured the secretion of IL-8 by DTT-treated Del-N35TCTP, BMOE or BM(PEO)_3_-treated RrTCTP, and BMOE or BM(PEO)_3_-treated DTT-pretreated-Del-N35TCTP (Figure 5B). Chemical crosslinkers did not influence RrTCTP. The activity of the DTT-treated Del-N35TCTP was 71.7±4.4%, and the activities of crosslinked forms were 108.5±0.1% and 122.4±8.7% of that of DTT-untreated Del-N35TCTP, respectively.

Next, we questioned if dimerization or truncation is important for cytokine-like activity. To answer this question, it was necessary to test the activity of dimerized full-length TCTP, which was not formed by the chemical crosslinkers. Therefore, we generated a dimeric form of full-length human recombinant TCTP (HrTCTP) by fusing it to rabbit Fc region (Figure 5C). The C_H_3 of Fc region can form a non-covalent homodimer [27], [28]. We analyzed rabbit Fc protein (Fc) and Fc-fused HrTCTP by Western blotting using rabbit IgG specific antibody. The apparent molecular weight of the Fc-fusion proteins increased due to glycosylation at Fc region in denaturing gel (Figure 5D), and the proteins were detected at a position corresponding to the dimer in native gel (Figure 5E). The untruncated-full length protein (Fc-HrTCTP) was more active than HrTCTP in BEAS-2B cells (Figure 5F), suggesting that dimerization of TCTP, rather than truncation, is essential for the activation of TCTP in allergic responses.

### NH_2_-terminal truncated RrTCTP showed increased affinity to the putative receptor

**Figure 6:**
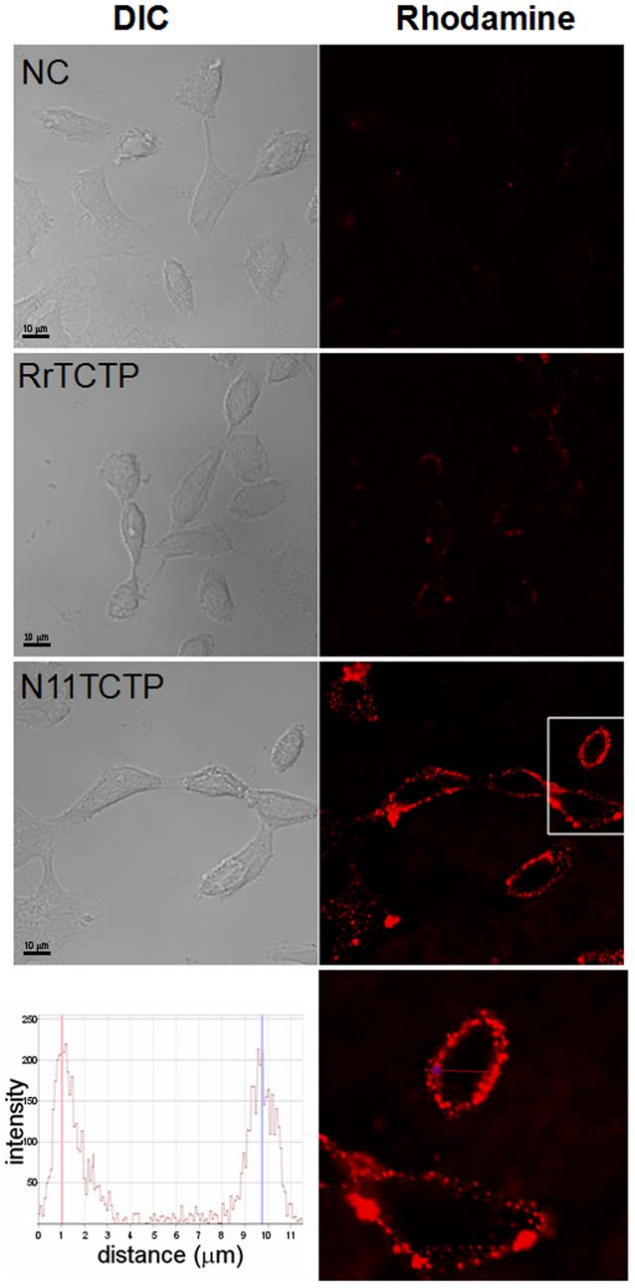
NH_2_-terminal truncated RrTCTP has strong affinity for the receptor in BEAS-2B cells. NC (without protein), RrTCTP, Del-N11TCTP were incubated with BEAS-2B cells at 4°C, and stained with rabbit anti-TCTP and rhodamine-conjugated goat anti-rabbit antibody. Confocal immunofluorescence (right) and corresponding DIC (left) images are presented (800X, 1600X for bottom panel). Scale bars indicate 10 µm.

We hypothesized that the conformational difference between RrTCTP and NH_2_-terminal truncated RrTCTP may lead to differential affinities to its receptor in plasma membrane. Although the TCTP receptor has not been identified, binding between TCTP and TCTP receptor has been demonstrated using FACS analysis in several reports [29], [30]. We used the confocal microscopy to evaluate the affinity of TCTPs to the receptor (Figure 6). When equivalent amounts (5 µM) of RrTCTP or Del-N11TCTP were incubated with BEAS-2B cells, and stained with rabbit anti-TCTP and rhodamine-conjugated goat anti-rabbit antibody, we found stronger fluorescence in plasma membrane of BEAS-2B cells incubated with Del-N11TCTP rather than with RrTCTP. The *z* stack analysis showed Del-N11TCTP was exclusively localized at the plasma membrane (bottom panel in Figure 6).

### NH_2_-terminal truncated RrTCTP promotes allergic airway inflammation in mice

**Figure 7:**
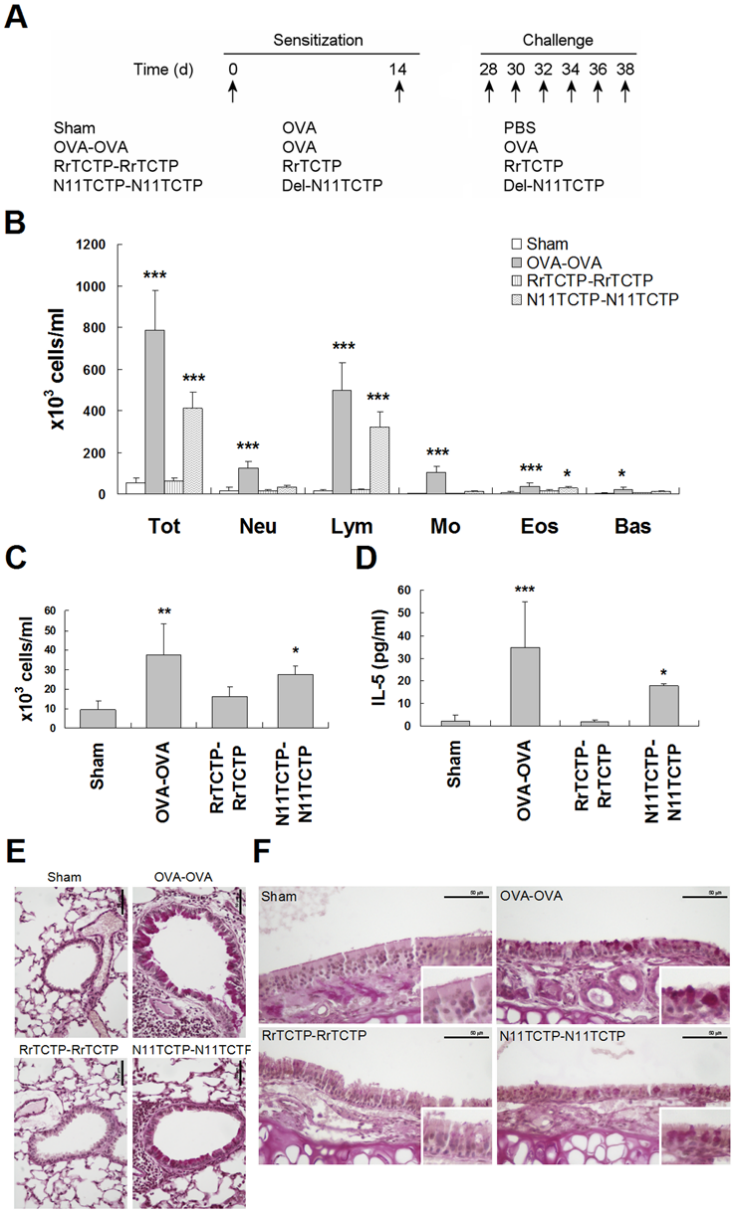
NH_2_-terminal truncated RrTCTP induces airway inflammation. (A) Six groups of mice were sensitized at days 0 and 14, and were challenged at days 28–38. (B, C) Total BALF cells and leukocytes in BALF were counted using HEMAVET HV950FS (Drew Scientific Inc.) at day 40. The eosinophils count (C) was re-displayed with small scale (* *p*<0.05, **p<0.01, ***p<0.001, significantly different from the Sham value, n = 4–6). (D) The IL-5 level in BALF was analyzed by mouse IL-5 ELISA kit (PIERCE) (* *p*<0.05, *** *p*<0.001, significantly different from the Sham value, n = 4–6). (E, F) Lung tissue (E) and nasal mucosa section (F) were prepared at day 40, and stained with periodic acid-Schiff (PAS) reagent. Mucus containing goblet cells were showed as deep purple. Scale bars indicate 50 µm.

Allergenic activity of Del-N11TCTP prompted us to see whether the NH_2_-terminal truncated TCTP can induce allergic airway inflammation *in vivo*. We sensitized and challenged BALB/c mice with either RrTCTP or Del-N11TCTP and examined the development of allergic lung inflammation compared to sensitization and challenge with OVA (Figure 7A). To evaluate the severity of inflammatory response, the number of BAL cells in total BALF was counted in a hemocytometer (Figure 7B). Total number of immune cells in BALF was significantly increased by OVA injection. Whereas RrTCTP injection (RrTCTP-RrTCTP) had no effect on immune cell infiltration, Del-N11TCTP injection (N11TCTP-N11TCTP) showed increases in total number of cells in BALF, in particular lymphocytes and eosinophils (Figure 7, B and C). In addition, IL-5 level in BALF was markedly increased by Del-N11TCTP (Figure 7D), reflecting the allergenic activity of Del-N11TCTP *in vivo*. Because accumulation of eosinophils in airway tissue contributes to mucus production [31], we sectioned and stained lung and nasal mucosa with PAS. As expected, OVA injection induced goblet cell hyperplasia in the lung and nasal mucosa (Figure 7, E and F). While RrTCTP had no influence on airway inflammation, Del-N11TCTP increased goblet cell hyperplasia in both lung and nasal mucosa, suggesting that NH_2_-terminal truncated TCTP, rather than full length TCTP, is an active form of TCTP that can induce allergic airway inflammation.

### Dimerized TCTP was identified in serum and BALF

**Figure 8:**
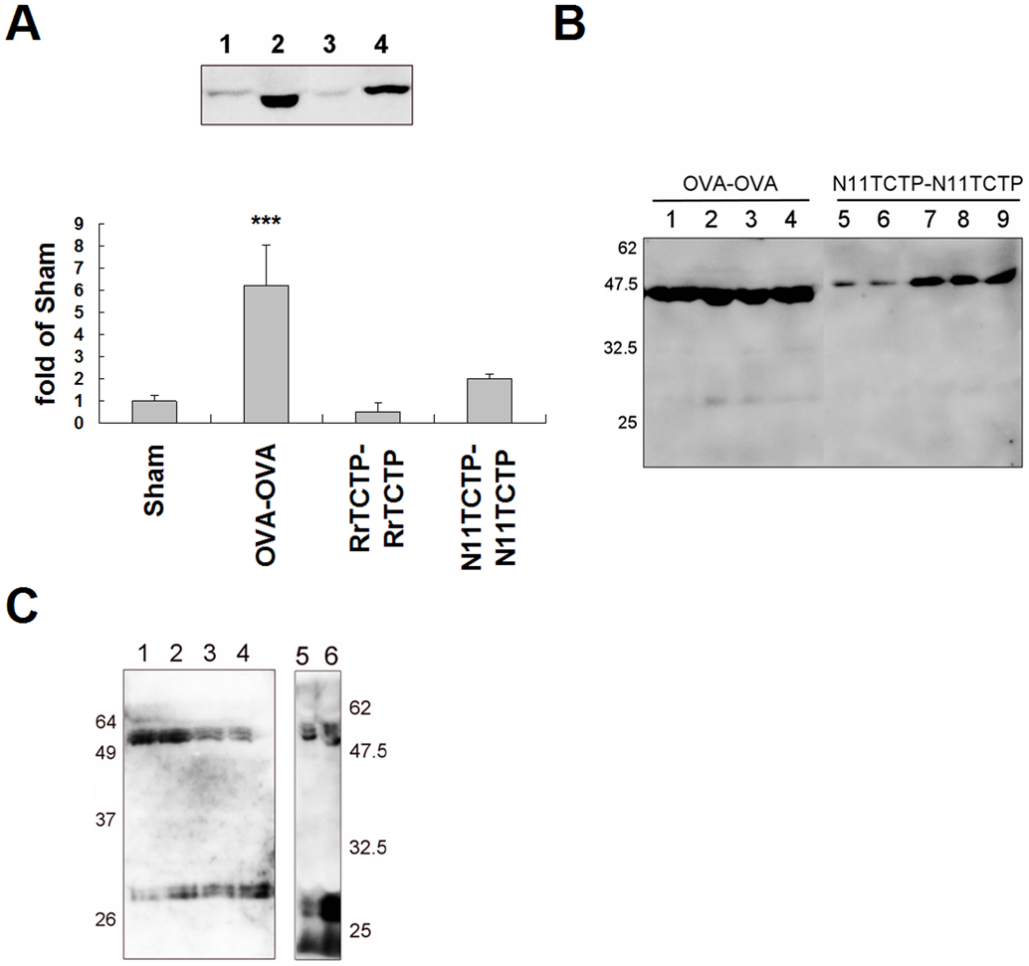
TCTP exists as dimers in allergic fluids. (A) 50µl of BALFs were analyzed by non-reducing SDS-PAGE and Western blotting. The present figure is representing 4 results. The intensity of each band was quantified by Multi Guage software (Fujifilm). (B) The BALF from OVA-OVA (OVA sensitized and OVA challenged, lane 1–4), and N11TCTP-N11TCTP (Del-N11TCTP sensitized and Del-N11TCTP challenged, lane 5–9) groups were analyzed by non-reducing SDS-PAGE and Western blotting. (C) The sera from atopic (lane 1–4) or atopic/asthmatic (lane 5, 6) patients were analyzed by non-reducing SDS-PAGE and Western blotting.

OVA challenge is known to stimulate the secretion of mouse TCTP and infiltration of eosinophils in an OVA-immunized mice model [32]. Since development of airway inflammation is observed in OVA-OVA and N11TCTP-N11TCTP sensitization/challenge experiments, we examined the relation between the severity of allergy and TCTP secretion to the BALF in the same model. Although various amounts of TCTP were found secreted in all the groups, only the mice suffering from inflammation showed increased TCTP secretion (Figure 8A). We also detected dimerized TCTP in BALFs from mice with airway inflammation (Figure 8B). Interestingly, almost all TCTP in BALF was detected at a position corresponding to the dimer in non-reducing SDS-PAGE.

Next, we tried to detect dimerized TCTP in serum of atopic and atopic/asthmatic patients. We could not detect the dimerized TCTP in serum denatured in sample buffer (50 mM Tris, pH 6.8, 2.5% SDS, 22% glycerol). We, therefore, precipitated the serum proteins with acetone/TCA [33] and denatured them in 8 M urea/2% CHAPS (Figure 8C). TCTP was detected in both monomer and dimer size, and these were not single form in non-reducing SDS-PAGE.

## Discussion

Our data suggest that NH_2_-terminal truncated RrTCTPs potently stimulate inflammatory responses. Del-N11TCTP and Del-N35TCTP at low concentration (1 µm/ml) could stimulate IL-8 release, but RrTCTP at the same concentration did not induce measurable IL-8 release. This activity was repeatedly observed in GM-CSF, IL-2, and IL-4 release and in airway inflamed mouse model. Thus, it would appear that some NH_2_-terminal truncated forms e.g. Del-N11TCTP or Del-N35TCTP might play a major role in allergic response *in vivo*. We also showed that the dimerization of NH_2_-terminal truncated TCTPs via intermolecular disulfide was a key factor to the activation of TCTP. According to the solution structure of TCTP from *Schizosacchromyces pombe* [34], the NH_2_-terminus and the COOH-terminus of TCTP are packed as antiparallel β-sheets. Thus part of the NH_2_-terminus may prevent disulfide bond formation. We found truncation of at least four amino acids (residues 1–4) is required to dimerize RrTCTP (data not shown). When the NH_2_-terminus is removed, Cys^172^ becomes exposed, and TCTP is able to form intermolecular disulfide bond.

Extracellular TCTP has been identified in the culture supernatants of the human U937 macrophage cell line [1], and several stimulators, such as H_2_O_2_ [12], M-CSF [32], and dioxin [35] have been found to promote the secretion of TCTP homologues. Teshima *et al*. proposed that the 30 kDa protein detected in the supernatant of OVA-elicited macrophages was probably an N-glycosylated mouse TCTP, since this protein treated with N-glycosidase F migrated to the same position as that in the cytosol [32]. However, since intraperitoneal injection of mouse recombinant TCTP that was not glycosylated could induce eosinophil migration in OVA-immunized mice, but not in normal mice [32], N-glycosylation may not be necessary for the cytokine-like activity of mouse TCTP. Moreover, a recent report showed that the secretion of human TCTP is not limited to cells that are activated by stimulators. These human TCTPs have the same apparent molecular weight both in the supernatants and in cell lysates of unstimulated embryonic kidney cells (293T), hepatocytes (HepG2), and erythroleukemia cells (K562) [36]. These findings suggest that human TCTP may be present in normal serum without causing any allergic syndrome and therefore, the cytokine-like activity of TCTP homologues may be due to a modified form that is generated in allergic environments.

MacDonald et al. showed that extracellular TCTP purified from culture supernatants of U937 cells contains the NH_2_-terminal sequence [1]. We examined TCTP secreted from U937 cells stimulated by M-CSF as well as H_2_O_2_ and detected only monomers (data not shown). However, we could identify TCTP dimers in sera of allergic patients and BALF of allergic mouse, suggesting that allergic symptoms caused by TCTP may occur only if TCTP is modified in allergic environment. The modification may be caused by oxidants released by neutrophils at the site of inflammation [37], or truncation by endogenous proteases from neutrophils [17], mast cells [18], and cytotoxic T cells [38] or exogenous proteases originating from the house dust mites *Dermatophagoides pteronyssinus* [39] and *Dermatophagoides farinae* [40]. Therefore, it is possible that the reported cytokine-like activity of TCTP might be due to the presence of a partially cleaved TCTP. Indeed, there was a faint band of partially cleaved RrTCTP in the absence of H_2_O_2_, and a faint band of dimer in the presence of H_2_O_2_ (Figure 3E and 5A), suggesting that full-length rTCTP purified from E. coli was partially digested probably by contaminating proteases. Alternatively, other forms of active TCTP e.g. TCTP bound by a TCTP-specific IgE [41], may exist and the NH_2_-terminal deleted proteins observed in our study might possess the active conformation fortuitously. Studies on TCTP homologues identified from Parasites [24], [42] suggest the possibility of presence of autoantibodies. Indeed, our experiment with Fc-HrTCTP, in which TCTP was fused to Fc region of antibody and dimerized, suggest the possible modification of TCTP by antibodies *in vivo*.

We also showed that dimerized TCTP exists in sera of atopic and atopic/asthmatic patients and in BALFs from mice with airway inflammation. Interestingly, the TCTP in BALF was difficult to convert into a monomer in reducing sample buffer, which suggests the existence of strong non-covalent bond(s). We also detected the dimer in C172S mutant and incomplete loss of activity of DTT-treated Del-N35TCTP, suggesting that the dimeric form still remained after the disruption of disulfide bond by Cys^172^. Based on their observation that *Brugia malayi* TCTP (BmTCTP) forms a dimer under non-reducing conditions, whereas monomeric form is detected predominantly under reducing conditions, Gnanasekar et al. proposed self-interaction by Lupas coiled coil structure of residues 92–124 in BmTCTP [24]. Therefore, it is possible that other interactions such as Lupas coiled coil structure might be also involved in the formation of TCTP dimer. However, our results strongly suggest that dimerized TCTP is the active form *in vivo* rather than the intact monomer. We propose that the TCTP-specific receptor can be possibly identified using the dimer form of TCTP, and this possibility is under investigation.

In conclusion, this study suggests that there are conformational differences between full-length RrTCTP and NH_2_-terminal truncated RrTCTP, and only the dimerized RrTCTP possesses cytokine-like activity.

## Materials and Methods

### Preparation of RrTCTP, truncated TCTPs, and mutated TCTPs

The sequences coding rat TCTP and deleted rat TCTPs (RrTCTP, residue 1–172; Del-N11TCTP, residues 11–172; Del-N35TCTP, residues 35–172; Del-N39C110TCTP, residues 39–110) were amplified using the oligonucleotide primers for cloning into the bacterial expression vector, pRSET A (Invitrogen). The residues 1–172 was amplified using forward-CG GGATCC (*Bam*HI) ATG ATC ATC TAC CGG GAC- and reverse primer-CCC AAGCTT (*Hind*III) ACA TTT TTC CAT CTC TAA GCC, the residues 11–172 was amplified using forward-CG GGATCC (*Bam*HI) GAC GAG CTG TCC TCC GAC AT- and reverse primer-CCC AAGCTT (*Hind*III) ACA TTT TTC CAT CTC TAA, the residues 35–172 was amplified using forward-CG GGATCC (*BamH*I) AGT GTC AGT AGA ACA GAG- and reverse primer-CCC AAGCTT (*Hind*III) ACA TTT TTC CAT CTC TAA, and the residues 39–110 was amplified using forward-CG GGATCC (*BamH*I) ACA GAG GGT GCC ATC GA and reverse primer-G GAATTC (*EcoR* I) CCT TTC TGG TTT CTG TT.

Three mutations were introduced into the Del-N11TCTP (Del-N11TCTP-C28S and Del-N11TCTP-C172S) and Del-N35TCTP (Del-N35TCTP-C172S) in order to eliminate the cysteine residues using the QuickChange Site-Directed Mutagenesis Kit (Stratagene). The pRSET A/Del-N11TCTP and pRSET A/Del-N35TCTP were amplified with complimentary oligonucleotide primers containing the mutation; for Del-N11TCTP-C28S, CG GAC GGG CTG TCT CTG GAG GTG GA and TC CAC CTC CAG AGA CAG CCC GTC CG, for Del-N11TCTP-C172S and Del-N35TCTP-C172S, GAG ATG GAA AAA TCT AAG CTT GAT CCG and CGG ATC AAG CTT AGA TTT TTC CAT CTC.

*Escherichia coli* BL21(DE3)pLysS cells transformed with the pRSET A/TCTPs or pRSET A/mutated TCTPs were overexpressed and purified using a Ni^2+^-charged His-Bind column according to manufacturer's protocol (Novagen). The NH_2_-terminal fusion proteins were separated by fast protein liquid chromatography (FPLC) on a Mono Q HR 5/5 column (Amersham Pharmacia Biotech) using a NaCl gradient. Buffer A was 20 mM Tris-HCl, 1 mM EDTA, 50 mM NaCl, pH 7.4, and Buffer B was 20 mM Tris-HCl, 1 mM EDTA, 1 M NaCl, pH 7.4. A linear gradient from 0% to 15% Buffer B was run over 5 column volumes and it was followed by a linear gradient from 15% to 50% Buffer B over 25 column volumes to elute the TCTPs. Each eluted fraction was desalted and transferred into PBS using PD-10 column (Amersham Pharmacia Biotech). Final proteins were detected LPS contaminations by the Limulus Amebocyte lysate (LAL) assay (Cambrex). The LAL assay showed that LPS contaminations ranged from 1 to 100 pg/µg protein. Because it had been known that BEAS-2B cells were relatively insensitive to LPS [12], proteins purified using FPLC were used in cytokine assays without further purification. The purity of the proteins was 85–98%. For T cells and sensitization and challenge of mice airway, proteins were additionally purified with EndoTrap (Cambrex).

### Measurement of IL-8 and GM-CSF

Human bronchial epithelial cells, BEAS-2B (ATCC), were maintained in bronchial epithelial growth medium (BEGM, Clonetics). When BEAS-2B cells became 80% confluent, they were passaged in 48-well culture plates, incubated for 20–24 h in BEGM, washing twice with 1% penicillin-streptomycin/bronchial epithelial basal medium (BEBM), and then incubated for the indicated time with 1–10 µm/ml of proteins or without protein (negative control, NC) in 1% penicillin-streptomycin/BEBM. IL-8 and GM-CSF secreted into media were measured by ELISA using a commercial kit (PIERCE).

### Measurement of IL-2

Human jurkat T cells (ATCC) were maintained in RPMI 1640 containing, 10 mM HEPES, 2 mM L-Glutamine, 1 mM sodium pyruvate, 1.5 g/L sodium bicarbonate, 4.5 g/L glucose, 10% heat-inactivated FBS, 1% penicillin-streptomycin. Cells were preincubated at 10^6^ cells/well for 4 h with TCTPs in growth medium or with medium alone (NC), and then stimulated with 10 µm/ml PMA and 25–500 µm/ml ionomycin for 20 h. IL-2 in supernatant was measured using ELISA kit (PIERCE).

### In vitro activation and differentiation of T_H_ cells

CD4^+^ T_H_ cells were isolated from the draining lymph node of the C57BL6 mice using mouse CD4 microbeads according to the manufacturer's instruction (Miltenyi Biotec). Cells were stimulated with anti-CD3 (1 µg/ml) and anti-CD28 (1 µg/ml) antibodies (BD Pharmingen) for 48 h, or restimulated with PMA (10 µm/ml) and ionomycin (1 µM) at 72 h. For T_H_2 cell differentiation, cells were co-stimulated with IL-4 (10 µm/ml) and anti-IFN-γ Ab (5 µg/ml) at day 1. Cells were induced to differentiate for additional 5 days and re-stimulated with anti-CD3 for 24 h. CD4^+^ T_H_ cells were incubated with RrTCTP or Del-N11TCTP accord with TCR stimulation. Supernatants were harvested at 48 h or at 24 h after secondary PMA/ionomycin or anti-CD3 stimulation. Supernatants were incubated with capturing antibodies specific for IL-2, IL-4, or IFN-γ, and subsequently biotinylated anti-cytokine antibodies as instructed (BD Pharmingen).

### Intracellular cytokine staining

CD4^+^ T_H_ cells were stimulated with anti-CD3 and anti-CD28 antibodies in the presence of RrTCTP or Del-N11TCTP for 72 h and additionally stimulated with PMA and ionomycin for 4 h. Cells were treated with Monensin (Sigma-Aldrich) for 2 h before harvest and subsequently incubated with phycoerythrin (PE)-tagged anti-IL-2 Ab or allophycocyanin (APC)-conjugated IL-4 Ab. After washing the cells with PBS, cells were analyzed by FACS Calibur and CellQuest program (BD Biosciences).

### Development of airway inflammation and lung analyses

BALB/c mice were sensitized at days 0 and 14 (i.p.) with OVA, RrTCTP or Del-N11TCTP. At day 28, the sensitized mice were challenged with the same allergen (300 µg of OVA, 10 µg of RrTCTP or 10 µg of Del-N11TCTP) by intranasal administration six times. At day 40, the mice were sacrificed and lungs and bronchoalveolar lavage (BAL) fluids were collected and immune cells were counted from the cytocentifuged cells. The supernatant was used for IL-5 measurement by ELISA (PIERCE) and TCTP detection by Western blotting. Lungs and rhinal tissues were fixed in 4% paraformaldehyde. After dehydration in alcohol, tissues were embedded in paraffin and cut into 5 µm thick sections. Tissue sections were stained with PAS (Periodic Acid Schiff) solution (Sigma-Aldrich). All animals were handled in strict accordance with good animal practice as defined by the relevant national and/or local animal welfare bodies, and all animal work was approved by Ewha Womans University's institutional animal care and use committee.

### Detection of the dimerized TCTP in sera of atopic and atopic/asthmatic patients

All the subjects enrolled in this study gave written informed consent, and the study protocol was approved by the Institutional review board of Seoul National University Hospital (No. 0706-040-211). The serum was precipitated with acetone/TCA, denatured in 8 M urea/2% CHAPS, and analyzed on non-reducing SDS-PAGE.

### Confocal microscopy

BEAS-2B cells seeded on confocal dish (SPL) were blocked with 1% BSA/BEBM at RT for 10 min prior to 30 min incubation with 5 µM of RrTCTP or Del-N11TCTP at 4°C. NC (without protein), RrTCTP, Del-N11TCTP treated cells were washed and labeled with rabbit anti-TCTP and rhodamine-conjugated goat anti-rabbit antibody (Jackson ImmunoResearch) at 4°C. The cells washed and then fixed in 3.7% formaldehyde/PBS for 15 min. Confocal microscopy was performed using a Carl Zeiss Laser Scanning Systems LSM 510. Z-stack images were taken at 0.1-µm intervals, and the profile was analyzed.

### Chemical Cross-linking of RrTCTPs

RrTCTP and Del-N35TCTP were irreversibly cross-linked by a crosslinker for primary amines, DMS (dimethyl suberimidate· 2HCl, PIERCE), and two crosslinkers for sulfhydryl groups having different lengths of spacer arms, BMOE (bis-maleimidoethane, PIERCE), and BM(PEO)_3_ (1, 11-bismaleimidotriethylene glycol, PIERCE). FPLC purified proteins were incubated with thirty-fold molar excess of DMS for 30 min, five-fold molar excess of BMOE for 2 h, or five-fold molar excess of BM(PEO)_3_ for 1 h at room temperature. The reaction of DMS was stopped by adding Tris, pH 7.5 at 50 mM final concentration; in all reactions, the excess nonreacted crosslinkers was removed by vivaspin ultrafiltration spin column (Vivascience). The resulting products were analyzed on SDS-PAGE, and assessed the IL-8 secretion activity in BEAS-2B cells.

### Overexpression and purification of HrTCTP and its variants

pCEP4 expression vector (Invitrogen) was modified to facilitate the construction of rabbit Fc fusion proteins by fusing sequences encoding HrTCTP and its variants, Del-N11HrTCTP and Del-N35HrTCTP, to the Fc region of rabbit IgG heavy chain. Human IgG kappa chain leader sequence and the rabbit Fc were linked by overlap extension PCR and the product was cloned in between the *Hin*dIII and *Bam*HI sites of the pCEP4 vector. Three cysteines in the hinge region of the rabbit Fc region were converted to serine. The TCTP and its variants were cloned in *Sfi*I site between the leader sequence and the rabbit Fc region. Full length human TCTP gene was amplified by using a sense primer with *Sfi*I site ( GGCCCAGGCGGCC ATG ATT ATC TAC CGG GAC CTC ATC AGC C) and an anti-sense primer with *Sfi*I site ( GGCCGGCCTGGCC ACA TTT TTC CAT TTC TAA ACC ATC CTT AAA GAA AAT CAT ATA TGG GGT CAC ACC ATC C). This anti-sense primer was also used for amplifying Del-N11HrTCTP and Del-N35HrTCTP genes. A sense primer with *Sfi*I site for Del-N11HRF gene was GGCCCAGGCGGCC GAT GAG ATG TTC TCC GAC ATC TAC AAG ATC CGG G and for Del-N35HRF gene was GGCCCAGGCGGCC ATG GTC AGT AGG ACA GAA GGT AAC ATT G.

The constructs were transfected into HEK293T cells, and the culture supernatant was harvested and clarified by centrifugation at 4,000 rpm for 30 min and passed through a bottle-top filter (Millipore Corporation) before application to a protein A-agarose column (Repligen). After washing with binding buffer (PIERCE), the binding proteins were eluted with 0.1 M glycine-HCl (pH 2.2). The eluate was neutralized with 1 M Tris (pH 9.5). Protein concentration was measured by BCA assay kit (PIERCE).

### Statistical analysis

Results are expressed as means±SD. We evaluated the results with a one-way ANOVA followed by multiple-comparison tests. Differences were considered significant if *p* values were<0.05. The Tukey's HSD test is applied to all pairwise differences between means, and Dunnett's test was performed to compare each group with the control.
